# Factors associated with suicide in physicians: a silent stigma and public health problem that has not been studied in depth

**DOI:** 10.3389/fpsyt.2023.1222972

**Published:** 2023-08-14

**Authors:** Narda Katherine Rátiva Hernández, Tania Yaritza Carrero-Barragán, Andrés Felipe Ardila, Juan Diego Rodríguez-Salazar, Ivan David Lozada-Martinez, Elisa Velez-Jaramillo, Daniel Aicardo Ortega Delgado, Ornella Fiorillo Moreno, Elkin Navarro Quiroz

**Affiliations:** ^1^Research Department, Department of Medicine and Psychiatry, Universidad Libre, Cali, Colombia; ^2^Department of Psychiatry, Universidad Autónoma de Bucaramanga, Bucaramanga, Colombia; ^3^School of Medicine, Universidad Autónoma de Bucaramanga, Bucaramanga, Colombia; ^4^Department of Medicine, Jacobi Medical Center–Albert Einstein College of Medicine, New York, NY, United States; ^5^Epidemiology Program, Department of Graduate Studies in Health Sciences, Universidad Autónoma de Bucaramanga, Bucaramanga, Colombia; ^6^Department of Medicine, Universidad Cooperativa de Colombia, Medellin, Colombia; ^7^Clinica Iberoamérica, Barranquilla, Colombia; ^8^Clinica El Carmen, Barranquilla, Colombia; ^9^Life Science Research Center, Universidad Simon Bolivar, Barranquilla, Colombia

**Keywords:** suicide, physicians, health personnel, mental health, social stigma, suicide prevention

## Abstract

Suicide is a complex and multifaceted public health issue that affects individuals from all walks of life, including healthcare professionals such as physicians. According to research, physicians have a higher risk of suicide compared to the general population, with an estimated suicide rate that is two to three times greater than that of the general population. Suicide in physicians can have devastating consequences, not only for the individual but also for their patients and colleagues. The factors contributing to suicide in physicians are numerous and often interrelated. Physicians are exposed to numerous stressors in their daily lives, including long work hours, high workload, burnout, and exposure to traumatic events. These stressors can lead to mental health problems such as depression, anxiety, and substance use disorders, which in turn can increase the risk of suicide. In addition to work-related stressors, personal factors such as relationship problems, financial stress, and a history of mental health problems can also contribute to suicide risk in physicians. Stigma and shame around seeking help for mental health issues may also prevent physicians from seeking treatment, exacerbating the problem. Understanding the complex factors that contribute to suicide in physicians is crucial for developing effective prevention strategies. For this reason, it is necessary to know the behavior of this phenomenon and the factors associated with a higher risk of suicide in this population. However, taking into account that different regions of the world vary in socioeconomic, cultural, professional, occupational, and health attributes, it is to be expected that the behavior of these risk factors will also be heterogeneous. At present, it is presumed that there is a significant gap in the evidence, due to a predominance of evidence on this topic from high-income countries. Considering the importance of having a comprehensive understanding of the risk factors for suicide in the medical population and possible strategies to mitigate this condition, the aim of this review is to analyze the most recent evidence on these factors, and to assess the quality of the evidence and gaps that need to be studied further.

## 1. Introduction

Currently, mental health is one of the sustainable development goals, and it is a necessary determinant for a human being to be considered healthy (1). Population transition, social and economic dynamics, restructuring of stereotypes and search for acceptance, emerging diseases, among other factors, significantly influence the mental state (2), making it susceptible to alteration and triggering a clinical spectrum that can generate morbidity, alteration of functional capacity and quality of life, and even death. One of the most dangerous and significant final manifestations and conditions is suicidal ideation and suicide (3).

Suicide is a significant problem in public health and global health (4). It is a tragedy that substantially impacts the emotional stability of family members, friends, colleagues, and other scenarios (5). Suicidal ideation, which often precedes suicide, represents a significant challenge for clinical and social management, as there is no consensus on its definition and clinical spectrum (3). However, it is evident that these ideas represent a warning sign of the presence of a neurocognitive disorder that may be associated with numerous risk factors, which can be intensified or mitigated depending on the intimate support network and specialized care for the affected person (2, 5). Unfortunately, there is still social stigma around having a mental disorder, which represents a barrier to accessing timely specialized care and reducing the risk of complications. Some groups are exposed to a greater number of scenarios with the potential to alter emotional state, such as physicians (6, 7).

Although the working scenario for physicians varies across different regions of the world, generally, this is one of the professions that is most exposed to long working hours, a high academic workload, a lifestyle dependent on time availability, low salaries, lack of guarantees of fair contract access, disorders related to alcohol and drug consumption, exposure to various emotions triggered by approaching frustration, fear, illness, death, among others (8–10). Specifically, due to social and work stigma, physicians tend not to seek psychological or psychiatric assistance in case of presenting suicidal ideation or a mental disorder (6, 7), increasing the risk of suicide due to symptom intensification by the lifestyle (The intensification is a result of the persistence of stressors, which can be accompanied by burnout occurring at the physician’s work, personal, or academic level, leading to the onset of a psychiatric disorder). Recent evidence has reported that the prevalence of mental disorders in physicians is high, ranging from 37–67%, which increased even more from the coronavirus disease 2019 (COVID-19) pandemic (11, 12). Despite the above, there are significant gaps in the evidence on the specific prevalence of suicidal ideation and suicide in physicians, and especially what factors are associated with these manifestations or suicide prognosis (13–15), which allow the development of strategies and policies to mitigate the appearance and evolution of these manifestations, and reduce the burden of silent disease. Addressing the issue of physician suicide requires a multifaceted approach that involves addressing both personal and work-related stressors, reducing stigma around seeking help for mental health issues, and providing adequate support and resources to physicians who are struggling with mental health problems (6, 7). By working together, healthcare organizations, policymakers, and healthcare professionals can help to reduce the risk of suicide in physicians and promote the well-being of healthcare professionals. For all the above, this condition is considered a silent killer in physicians.

In search of providing relevant evidence that serves for the understanding of this condition, the aim of this narrative review is to evaluate the epidemiology, associated factors, quality of evidence and propose possible strategies for the control of suicide in physicians.

## 2. Epidemiology of mental disorders, suicidal ideation, and suicide in physicians

Knowing the distribution of a disease is as important as its risk factors in determining causality or predicting outcomes. Reporting on the prevalence of suicidal ideation, associated symptoms, basic characteristics, and physician suicide allows us to understand the physical and mental context of this group of professionals, who often face highly stressful situations. Despite this, there are very few studies that have investigated this phenomenon, and there are regions of the world where there are no up-to-date data on this condition.

In the early 2000s, researchers published an analysis of suicide in England from 1979 to 1995. The analysis reported that 223 physicians died by suicide, resulting in annual rates of 19.2 and 18.8 per 100,000 inhabitants for male and female physicians, respectively. At that time, the data showed that certain specialties, such as general practitioners, psychiatrists, community health doctors, and anesthesiologists, had a higher frequency of suicide (16). Approximately 10 years later, a group of Danish researchers utilized a national registry (based on data from 1981 to 2006) and found that healthcare providers, in general, had a higher risk of suicide compared to the general population. Specifically, physicians had an 87% higher risk of suicide (17).

In 2018, Vu-Eickmann et al. (18) analyzed 998 physicians in Germany to investigate the relationship between their psychological work environment and health outcomes, quality of life, and intention to leave the profession. They found that 73.7% of physicians reported suffering from work-related stress, and that those with work-related stress had a 3.6 to 8.83 times higher probability of experiencing negative health outcomes, high symptoms of depression and anxiety, committing more medical errors, and leaving the profession compared to those without work-related stress. Despite being conducted in high-income countries with better physical infrastructure, salary, and job opportunities for physicians, fewer than 5 studies were conducted within a nearly 20-year window of study, which reported high levels of neuropsychiatric symptoms (18). This suggests that there may have been unstudied inequities, or other associated factors had more impact on the physician’s work environment, such as workload, work infrastructure, and salary.

In Ireland, the BICDIS (Burnout in Consultants in Ireland Study) surveyed 477 physicians to determine the prevalence and covariates of burnout. The study revealed a prevalence of 42%, and demonstrated that burnout was associated with lack of exercise and face-to-face contact with patients, despite the physicians having good salaries and a relatively low workload (19). In a secondary analysis, Crudden et al. (20) found that approximately 26 and 14% of the study sample scored positively for depression and anxiety, respectively. They also showed that these symptoms were associated with high emotional exhaustion, reduced professional effectiveness, and increased work burden (*p* < 0.001) (20).

Meanwhile, in Spain, De la Vega Sánchez (21) evaluated the frequency of burnout and suicidal thoughts among 3,140 physicians during the COVID-19 pandemic. The study highlighted that up to 17.32% of physicians reported serious suicidal thoughts during this time, and this phenomenon was more common among women, those with a history of suicidal thoughts and psychiatric drug use, and those working in different areas. On the other hand, living with someone was a protective factor (21). These findings underscore the impact of social and family networks on the management of suicidal ideation and support for physicians with mental disorders. During the same period in New York (22), a survey of 225 physicians revealed that approximately 6 to 20% of them experienced burnout, depression, and suicidal ideation. Researchers found that these conditions were linked to a previous history of anxiety and depression. Suicidal ideation, in particular, was more common among younger physicians. Consequently, it is necessary to conduct a thorough study of the generational shift and new methods for coping with crises, as current methods may not be the most effective and may endanger the physical and emotional well-being of physicians and impact the dynamics of the healthcare team. This issue is more severe than previously thought because healthcare teams, who are expected to be well-equipped to handle crises, lack the necessary tools to do so effectively.

Between 1986 and 2020, 212 suicides were reported among physicians in Austria, with men accounting for the majority (69.3%) and the average age being around 50 years. Poisoning was found to be the leading cause of death within this occupational subgroup. It is worth noting that physicians had the highest suicide rate among a group of 7 occupations, which also included dentists, veterinarians, pharmacists, notaries, accountants, and lawyers (23). A recent online survey conducted in Germany evaluated 169 medical assistants responsible for various services such as surgery, internal medicine, emergency, and orthopedics during 2021. The results showed that 91.6% of the participants reported having favorable job satisfaction. Moreover, the survey revealed that job satisfaction was negatively associated with depression (*p* < 0.001), anxiety (*p* < 0.05), and stress (p < 0.05). However, the survey found a gender difference in stress level, which was reported to be higher among women (24). This finding was similar to the one reported by Irigoyen-Otiñano et al. (25) in Spain, who analyzed 11 years of physician suicide compared to the general population and showed that physicians have a higher risk of dying (1.3% vs. 0.8%; *p* = 0.003). However, female physicians had a 7.5% higher suicide rate compared to the general population, as well as a higher risk compared to male physicians (25).

All studies identified during this review that aimed to assess mental disorders, suicidal ideation, and suicide in physicians were conducted individually in Europe and the United States. Therefore, we can initially identify a significant knowledge gap and related factors regarding this phenomenon in physicians, which could help design a potential strategy to alleviate the emotional distress experienced by this subgroup. A meta-analysis that investigated the global prevalence of anxiety and depression in physicians during the COVID-19 pandemic, including 55 studies with over 60,000 responses, revealed that at the time, the prevalence of depression and anxiety was 20.5 and 25.8%, respectively (26). The American College of Cardiology conducted a global study to identify the prevalence and professional impact of mental conditions on 5,931 cardiologists, revealing that one in four cardiologists reported having a mental condition, mainly those from South America and Asia. Women were more likely to consider suicide in the last 12 months (3.8% vs. 2.1%) but were also more likely to seek help than men (42.3% vs. 31.1%; *p* < 0.001) (27). Thus, we observe different behaviors regarding these conditions between men and women, where the social stigma of being “weak” in suffering from a mental disorder may prevent physicians, especially men, from seeking mental help. Ultimately, this could be a potential risk factor for the final outcome, suicide.

**Figure 1:**
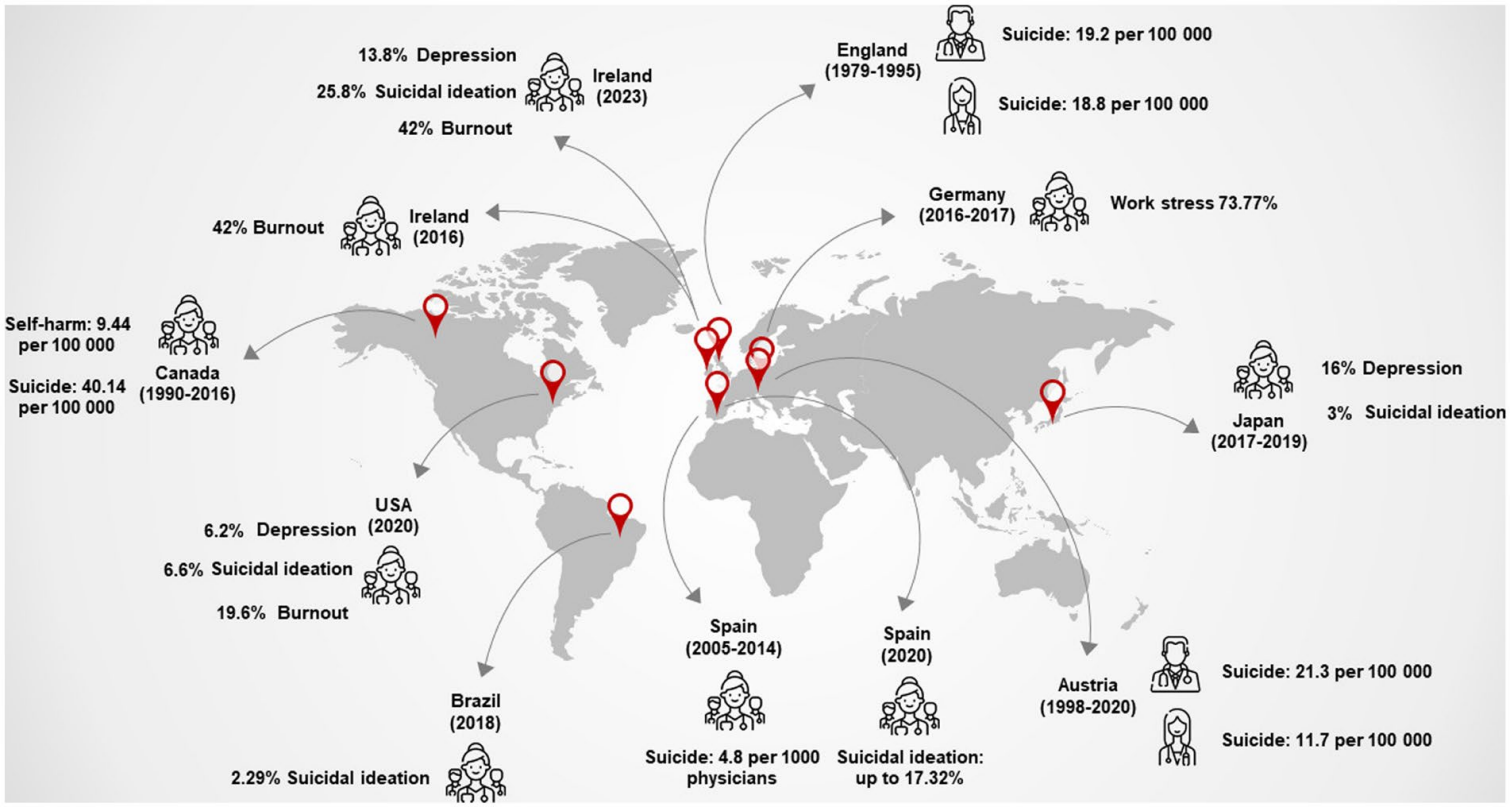
Prevalence and crude rates of psychiatric symptoms, suicidal ideation, self-harm, and suicide around the world among physicians (16–30). Data were distributed by sex, if this information was available (icons where sex is differentiated), and if not, the calculation was global. In the rates, the calculation is per 100,000 general populations or physicians at-risk per year. Authors.

Freire et al. (28) and Ishikawa (29) reported the prevalence of neuropsychiatric symptoms and suicidal ideation in Latin America and Asia, respectively, as secondary data. The prevalence of suicidal ideation ranged from 2.29 to 3%, and neuropsychiatric symptoms ranged from 12 to 16%. Associated factors such as marital status, age, workload, and type of hospital were heterogeneously identified (28, 29). Overall, the availability of updated data on the prevalence of neuropsychiatric manifestations, suicidal ideation, and suicide among different regions of the world (Figure 1) is clearly unequal, particularly in Latin America, Africa, and Asia. To comprehend the factors influencing these thoughts, support networks, and the final outcome, these data must be interpreted in the context of physicians’ working conditions, social context, and healthcare systems (30).

## 3. Risk factors for suicidal ideation and suicide in physicians

As of now, there are only a few studies that have specifically investigated the risk factors for suicidal ideation and suicide in physicians [Table 1; (28–33)].

**Table 1:** General characteristics of studies that have evaluated factors associated with suicidal ideation and suicide in physicians (28–33).

Authors	Number of participants *n*-Study Design Country	Risk factors/associated factors with suicidal ideation and suicide	Results	Conclusions
Freire et al. (28)	- *N* = 216 (*n* = 131; physicians) - Cross-sectional study - Brazil	- Marital status (without a partner) = PR 2.48; 95% CI: 1.36–4.53 - Previous suicide attempt = PR 8.91; 95% CI: 6.06–13.10 - Depression symptoms = PR 5.51; 95% CI: 3.17–9.57 - Anxiety symptoms = PR 3.08; 95% CI: 1.69–5. 63 - Stress symptoms = PR 5.53; 95% CI: 3.21–9.52	Overall, the prevalence of suicidal attempt among physicians was found to be 2.29%, although 15.28% reported a family history of suicidal ideation or suicide. The prevalence of symptoms of depression, anxiety and stress was 11.45, 14.50 and 16.03%, respectively. An association was found between marital status, presence of neuropsychiatric symptoms, sexual orientation, workload and previous suicide attempts with suicidal ideation	There are sociodemographic, familial and work-related factors associated with suicide risk among physicians. Also, a high prevalence of neuropsychiatric symptoms was found, as well as a high risk of suicide
Ishikawa (29)	- *N* = 919 (OB/GYNs) - Cross-sectional study - Japan	- Marital status (married): OR 0.41; 95% CI: 0.27–0.63 - Working hour per week (≥100): OR 2.08; 95% CI: 1.07–4.05	A higher prevalence of depressive symptoms and suicidal ideation was found in men, in physicians between 30 and 39 years of age, in non-university hospitals, and in those working more than 60 h per week	The frequency of suicidal ideation was more common among young male OB/GYNs who work in private institutions with a high workload
Sood et al. (30)	- *n* = 35,989 (physicians) - Cohort study - Canada	- Age (+ 45): OR 0.29; 95% CI: 0.09–0.95 (for suicide) / OR 0.20; 95% CI: 0.06–0.60 (for self-harm) - History of mood or anxiety disorder: OR 3.08; 95% CI: 1.22–7.79 (for suicide) / OR 2.84; 95% CI: 1.17–6.87 (for self-harm) - Any outpatient mental health visit in past year: OR 2.48; 95% CI: 1.05–5.83 (for suicide) / OR 3.08; 95% CI: 1.34–7.10 (for self-harm) - Any psychiatrist visits in past year: OR 3.79; 95% CI: 1.60–8.95 (for suicide) / OR 3.87; 95% CI: 1.67–8.95 (for self-harm)	The crude rate of self-harm and suicide among physicians was found to be 9.44 and 40.14 cases per 100,000 person-years at risk, respectively. Compared to the years 1990–2016, there was a higher frequency of self-harm or suicide in the period 2002–2016 (27 vs. 62 events). It was found that age, a history of anxiety disorder, and previous mental health consultation were associated with these outcomes	This study demonstrated that age, a history of psychiatric disorder, and previous consultation with a mental health service were associated with self-harm or suicide, and that the frequency of these outcomes has increased in recent years
Ji et al. (31)	- *N* = 767 (*n* = 485; physicians) - Cohort study - United States	- Ancestry (Asian or Pacific Islander) = OR 2.80; 95% CI: 1.96–3.99 - Job problems = OR 1.79; 95% CI: 1.49–2.17 - Civil legal problems = OR 1.61; 95% CI: 1.15–2.26 - Physical health problems = OR 1.40; 95% CI: 1.19–1.64 - Mental illness (currently receiving treatment): OR 1.45; 95% CI: 1.24–1.69 - Gender (female): OR 0.44; 95% CI: 0.35–0.55 - Marital status (unmarried): OR 0.36; 95% CI: 0.31–0.42	The probability of suicide varies when comparing surgeon vs. non-surgeon physicians, and compared to the general population. Surgeons are more likely to commit suicide if they are male, married, have mental illnesses under treatment, or if they have problems with alcohol or on the job. In non-surgical physicians, the work, social and health interaction network is a determinant in suicidal ideation or suicide	Several factors were found to be associated with and against suicide in non-surgical physicians and surgeons, who, compared to the general population, are more likely to suffer this type of outcome. The social, occupational and family support network is essential to prevent suicide in physicians
Dutheil et al. (32)	- Not determined - Meta-analysis of 31 studies - 11 countries	- Gender (female): SMR 1.9; 95% CI: 1.49–2.58 - Country (US physicians): ES 1.34; 95% CI: 1.28–1.55 - Region (Europe): ES -0.18; 95% CI: −0.37 to −0.01 - Medical specialties: ES 0.32; 95% CI: 0.21–0.43 (for general practitioners) /ES 0.16; 95% CI: 0.09–0.23 (for internal medicine) / ES 0.11; 95% CI 0.09–0.14 (for psychiatrists)	It was found that the prevalence of suicide among physicians is 14.4%, mainly in women, among American physicians (compared to other regions of the world), and in general practitioners, internists, and psychiatrists. Over time, a reduction in the prevalence of suicide was found among European physicians	The prevalence of suicide was higher in physicians compared to the general population, and there are gender and medical specialty disparities that may increase the risk of self-harm and suicide in physicians
Duarte et al. (33)	- Not determined - Meta-analysis of 13 studies - 6 countries	- Gender: SMR 1.46; 95% CI: 1.02–1.91 (for female) / SMR 0.67; 95% CI: 0.55–0.79 (for males)	It was found that there is a higher risk of suicide in female physicians compared to women in the general population. Conversely, the risk of suicide in male physicians is lower compared to men in the general population. Also, since 1980, there has been a decrease in suicide in both genders in medicine (SMR −0.84; 95% CI: −1.26 to −0.42 /SMR −1.96; 95% CI: −3.09 to −0.84)	A difference in the risk of suicide by gender among physicians was demonstrated, with a significantly higher risk in women, despite this trend having decreased in recent years

* ES, Effect-Size; PR, Prevalence Ratio; OB/GYNs, Obstetrician/Gynecologists; OR, Odds Ratio; SMR, Standardized Mortality Rate.

A study conducted in Brazil evaluated factors associated with suicide risk in physicians and nurses. It was found that, out of 131 physicians, suicidal ideation occurred in 12.2% (n = 16) of them, and there was a relatively high frequency of suicide attempts and successful cases of suicide among their family and friends (with a frequency ranging from 15.2 to 43.5%). Additionally, up to 16% of these physicians reported experiencing neuropsychiatric symptoms. The study revealed that factors such as not having a partner (Relative Risk [RR] 1.77; 95% CI: 1.02–3.09, *p* = 0.043), having a previous suicide attempt (RR 5.20; 95% CI: 2.49–10.88, *p* < 0.001), and experiencing stress (RR 2.71; 95% CI: 1.12–6.55, *p* = 0.027) or depression (RR 3.82; 95% CI: 1.70–8.55, *p* = 0.001) were associated with an increased probability of suicidal ideation or suicide (28). Meanwhile, a national survey was conducted in Japan by Ishikawa (29) to evaluate the mental health and suicidal ideation of 919 gynecologists/obstetricians. The study revealed that up to 16% of the participants had neuropsychiatric symptoms, 37% worked over 60 h per week, and 66% were aged between 30 and 50 years. The prevalence of suicidal ideation was 3% in the population, and a link was identified between working more than 100 h per week and a 600% (95% CI: 1.95–25.38; *p* < 0.001) increase in the likelihood of suicide, and up to 108% (95% CI: 1.07–4.05, *p* = 0.03) increase in the likelihood of depression. The study found that having a romantic partner reduced the likelihood of suicide and depression by 78% (95% CI: 0.08–0.56, p < 0.001) and 59% (95% CI: 0.27–0.63, p < 0.001), respectively. It is important to note that around 50% of the participants worked more than 80 h per week (29).

A large cohort from the College of Physicians and Surgeons of Ontario was analyzed by a group of researchers in Ontario, Canada, to identify self-harm and suicide in physicians. Similar frequencies (0.07% vs. 0.11%) and rates of suicides (9.44 vs. 11.55 per 100,000 person-years) were observed compared to the general population. Self-harm events requiring medical attention (Hazard Ratio [HR] 0.65; 95% CI: 0.52–0.82) or suicide (HR 0.70; 95% CI: 0.57–0.86) were less frequent in the medical community. A history of psychiatric disorder (Odds Ratio [OR] 2.84; 95% CI: 1.17–6.87), attendance to a mental health consultation the previous year (OR 3.08; 95% CI: 1.34–7.10), and attendance to a psychiatry consultation the same year (OR 3.87; 95% CI: 1.67–8.95) were associated with a higher likelihood of self-harm or suicide. Notably, psychiatrists had a up to 518% higher likelihood of suicide (1.78–21.53) (30). Furthermore, a cohort study conducted in the United States examined over 170,000 individuals who died by suicide to determine differences in frequency between healthcare providers and the general population. When healthcare providers were analyzed, 63.2% (*n* = 485) of suicides were non-surgeon physicians, while surgeons accounted for 13.4% (n = 103). The probability of suicide increased in the following order: 1) Older age (OR 1.003 per year; 95% CI: 1.002–1.004, *p* < 0.001); 2) Receiving psychiatric treatment (OR 1.32; 95% CI: 1.09–1.61; *p* = 0.005); 3) Physical health problems (OR 1.53; 95% CI: 1.25–1.87, p < 0.001); 4) Work-related problems (OR, 1.72; 95% CI, 1.35–2.20; *p* < 0.001); 5) Legal problems (OR 1.80; 95% CI: 1.19–2.71, *p* = 0.006); and 6) Ancestry (OR 2.92; 95% CI: 1.90–4.51, *p* < 0.001) (31). Compared with non-surgeon physicians, surgeons had a higher probability of receiving psychiatric treatment (up to 68%; 95% CI: 1.03–2.74, *p* = 0.04), but unmarried surgeons had a lower probability of dying by suicide (up to 51%; 95% CI: 0.29–0.83, *p* = 0.008) compared with non-surgeon physicians (31).

Recently, two meta-analyses were conducted to identify factors associated with suicide in general and among physicians based on gender. It was found that American physicians have a higher risk of suicide compared to the rest of the world (*p* < 0.001). Additionally, general practitioners (*p* < 0.001), internists (*p* < 0.001), and surgeons (*p* < 0.001) were found to have a higher risk of suicidal ideation or committing suicide worldwide (32). As for gender, the analyses revealed that female physicians have a higher risk of suicide compared to the general population (SMR [Standardized Mortality Ratio] 1.46; 95% CI: 1.02–1.91), while men have a lower risk compared to the general population (SMR 0.67; 95% CI: 0.55–0.79) (33).

This studies that have directly evaluated factors associated with suicidal ideation and suicide in physicians have highlighted several points for discussion. Firstly, these phenomena mainly affect adults aged approximately between 19 and 44 years, who are in their transition phase from junior to senior medical training, completing medical residencies or postgraduate studies that require a significant investment of time, and building their social, familial, and professional networks. This age group is particularly susceptible to emotional changes and requires a support system to mitigate the emotional and work burden experienced. This need is especially critical in the current world where humanity is exposed to countless stressful factors. Secondly, suicidal ideation is more frequent in physicians with high workloads, which can trigger frustration, particularly if accompanied by insufficient salaries and unfair employment contracts that demand constant physical and emotional effort. These factors can negatively impact the physician’s social or familial support networks and lead to isolation and loneliness. Also, there is a greater risk of suicidal ideation and suicide in physicians with a personal or family history of psychiatric disorders, suicidal ideation, or suicide. This constitutes a critical point regarding a possible unresolved health problem or grief that constantly attacks the physician’s mental health. Furthermore, physicians are exposed in their work setting to various inherent emotions of healthcare practice, such as confronting death, delivering bad news, and perceiving the pain and suffering of another human being. These factors may generate a pessimistic outlook on life and its development.

The high prevalence of unresolved neuropsychiatric symptoms in some regions and its implications for healthcare practice is an interesting point. Pereira-Lima et al. (34) conducted a meta-analysis that evaluated the association between depressive symptoms and medical errors, and found that this type of symptom increases the risk of committing a medical error by up to 95% (95% CI: 1.63–2.33). However, a deeper analysis reveals that the absence of institutional support and policies regulating the physician’s work environment, the agonizing lifestyle that physicians experience due to shortages of infrastructure, supplies, equipment, and lack of favorable perception from the community, as well as the social stigma that prevents physicians from seeking care and treatment from mental health services, can explain this prevalence. Surgeons, in particular, are at a higher risk of suicidal ideation, which is a common finding among the analyzed studies, given their lifestyle dependent on their work and the very high academic and work burden they face. Therefore, since some professionals are more exposed to psychiatric disorders than others, the approach, strategies, and incentives cannot be the same for all. Also, the role of the family and social support network is crucial, as it has been generally observed that married physicians have a lower prevalence of neuropsychiatric symptoms or suicidal ideation. However, this observation is subjective, as it depends on several factors such as the quality of the relationship, customs, among others. It could be related to the emotional support that physicians require to express their emotions.

The evidence shows that women are at higher risk of experiencing suicidal ideation or suicide. Unfortunately, physicians commonly report gender discrimination, which significantly impacts the affected person’s performance and quality of life. In the United Kingdom, Hussain et al. (35) qualitatively analyzed this phenomenon and found that affected individuals reported suffering from high levels of stress, fear, mistrust, low resilience, and retention. These microaggressions, harassment, or discrimination against women can be observed in regions with questionable medical professionalism, underestimating their potential and skills in practice. These behaviors can lead to frustration and negative emotions that culminate in psychiatric disorders, suicidal ideation, or suicide (36). Emotional burden and the impostor phenomenon are additional factors that may perpetuate and worsen symptoms over time. Previous studies have identified that up to 30% of residents experience these symptoms, and those who suffer from the impostor phenomenon have a probability of up to 113% (95% CI: 1.43–3.19) higher of experiencing burnout and, consequently, suicidal ideation (37).

It can be concluded that there are multiple factors, both favorable and unfavorable, associated with suicidal ideation and suicide in physicians that affect various items of their interaction network, including work, personal, family, and social domains. These findings provide a basis for proposing future strategies aimed at reducing the risk of suicidal ideation and suicide in physicians and mitigating the risk of medical errors.

## 4. Quality of evidence from studies evaluating factors associated with suicidal ideation and suicide in physicians

To evaluate the validity and value of the limited available evidence pertaining to the research question, we conducted a meta-research analysis focusing on the methodological quality of the study designs. The Joanna Briggs Institute checklists (38) were employed to assess the fulfillment of criteria for each of the six identified studies, which comprised two cross-sectional, two cohort, and two systematic reviews. Two authors independently evaluated the fulfillment of the criteria for each study design, and discrepancies were resolved by a third author, though such intervention was ultimately unnecessary.

To evaluate the risk of bias in each study design, we used relevant tools and guidelines (39–41) and established the following cut-off scores based on said guidelines: (1) a low risk of bias was indicated if 70% of the answers scored “yes”; (2) a moderate risk was indicated if 50 to 69% of the questions scored “yes”; and (3) a high risk of bias was indicated if the proportion of “yes” scores fell below 50% (38).

The analysis of the cross-sectional studies revealed that one had a high risk of bias (37.5% compliance), whereas the other had a low risk (75% compliance). The primary reason for the high risk of bias was the failure to comply with the requirement of describing confounding factors and strategies to mitigate their influence. In the study with the highest risk of bias, there was also a clear omission in the description of inclusion criteria, research setting, and subjects (Table 2).

**Table 2:** Methodological quality of cross-sectional studies that have evaluated factors associated with suicidal ideation and suicide in physicians.

Cross-sectional studies									
	Criterion #1	Criterion #2	Criterion #3	Criterion #4	Criterion #5	Criterion #6	Criterion #7	Criterion #8	Overall score
Freire et al. (28)	Yes	Yes	Yes	Yes	Unclear	Unclear	Yes	Yes	6/8
Ishikawa (30)	No	No	Yes	Yes	No	No	Yes	Unclear	3/8

* Criterion #1: Criteria for inclusion; Criterion #2: Study subjects and the setting; Criterion #3: Reliability and validity of exposure measurement; Criterion #4: Standard criteria used for measurement; Criterion #5: Confounding factors identified; Criterion #6: Strategies to deal with confounding factors; Criterion #7: Reliability and validity of outcomes measurement; Criterion #8: Appropriate statistical analysis.

The analysis of the cohort studies revealed a compliance rate of 81.8% for both studies, indicating a low risk of bias. However, criteria 4 and 5, which relate to the identification of confounding factors and strategies to mitigate them, were rated as “unclear” for both studies. This lack of clarity may impede the precision of outcome measurement. Nevertheless, based on our findings, it can be concluded that both studies possess good methodological quality (Table 3).

**Table 3:** Methodological quality of cohort studies that have evaluated factors associated with suicidal ideation and suicide in physicians.

Cohort studies												
	Criterion #1	Criterion #2	Criterion #3	Criterion #4	Criterion #5	Criterion #6	Criterion #7	Criterion #8	Criterion #9	Criterion #10	Criterion #11	Overall score
Sood et al. (31)	Yes	Yes	Yes	Unclear	Unclear	Yes	Yes	Yes	Yes	Yes	Yes	9/11
Ji et al. (29)	Yes	Yes	Yes	Unclear	Unclear	Yes	Yes	Yes	Yes	Yes	Yes	9/11

* Criterion #1: Recruitment and group similarity; Criterion #2: Similar measurement of exposure between the two groups; Criterion #3: Reliability and validity of exposure measurement.; Criterion #4: Confounding factors identified; Criterion #5: Strategies to deal with confounding factors; Criterion #6: Absence of exposure at the start of the study; Criterion #7: Reliability and validity of outcomes measurement; Criterion #8: Appropriate follow-up of outcomes; Criterion #9: Loss of follow-up; Criterion #10: Strategies to avoid incomplete follow-up; Criterion #11: Appropriate statistical analysis.

In the systematic reviews, one had a moderate risk (compliance rate of 63.6%), while the other had a low risk (compliance rate of 90.9%). Both reviews could be useful, although they should be interpreted with caution, given that all the studies evaluated the outcomes heterogeneously, which significantly affects the precision of the estimated effect. The criteria not fulfilled in these two systematic reviews consisted of methods to minimize data extraction error, assessment of risk of bias, lack of recommendations for policies and practices, and specific guidelines for future research (Table 4). Since this design represents the pinnacle of evidence, all criteria must be evaluated to obtain evidence of the highest quality, which can prevent delays in the development of evidence-based strategies, especially when the evidence is limited.

**Table 4:** Methodological quality of systematic reviews that have evaluated factors associated with suicidal ideation and suicide in physicians.

Systematic reviews												
	Criterion #1	Criterion #2	Criterion #3	Criterion #4	Criterion #5	Criterion #6	Criterion #7	Criterion #8	Criterion #9	Criterion #10	Criterion #11	Overall score
Dutheil et al. (32)	Yes	Yes	Yes	Yes	Yes	Yes	No	Yes	Unclear	Unclear	Unclear	7/11
Duarte et al. (33)	Yes	Yes	Yes	Yes	Yes	Yes	Yes	Yes	Yes	Unclear	Yes	10/11

* Criterion #1: Clarity of the review question; Criterion #2: Criteria for inclusion; Criterion #3: Search strategy.; Criterion #4: Sources and resources; Criterion #5: Criteria for appraising studies; Criterion #6: Critical appraisal conducted; Criterion #7: Methods to minimize errors in data extraction; Criterion #8: Methods used to combine studies; Criterion #9: Likelihood of publication bias; Criterion #10: Recommendations for policy and/or practice; Criterion #11: Specific directives for new research.

## 5. Possible strategies to be used to reduce mental health conditions in physicians

The burden of disease caused by mental conditions has been a significant focus for the World Health Organization, the United Nations, and the Pan American Health Organization (42–46). These organizations have highlighted the need to promote strategies that are cost-effective, cost-efficient, and easily replicable, and that can be accurately applied in this field. Mental well-being is a priority according to global health and sustainable development objectives, and all research and interventions aimed at preventing mental disorders are highly valuable (45, 46).

**Figure 2:**
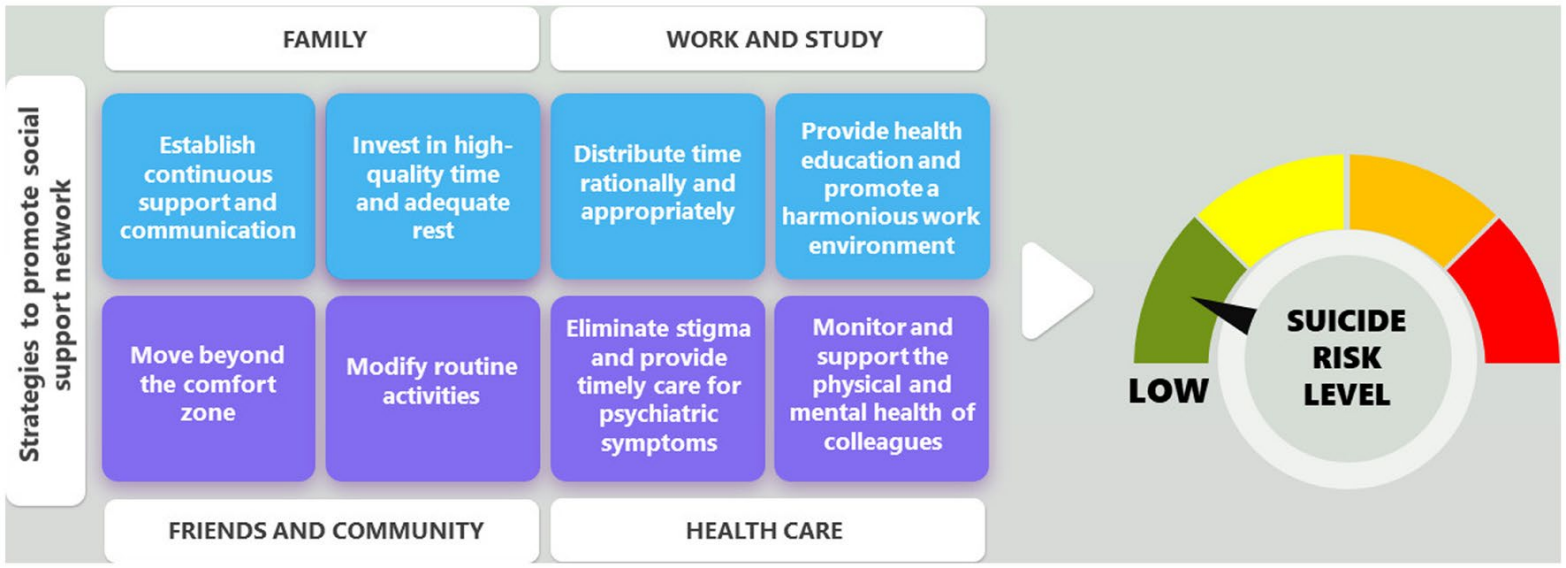
Possible strategies to implement in the social support network of physicians, in the domains of family, work and study, friends and community, and self-care, to ensure a very low or zero risk of suicidal ideation or suicide. Authors.

Based on available evidence, social support networks are indispensable for the physical and mental well-being of physicians **(**Figure 2). However, what sources of support and interventions exist in the literature that can promote resilience and mental health in physicians? Qualitative studies indicate that emotional support, equitable workload distribution, active and harmonious leadership, positive learning environments, anti-stigma activities, identification of vulnerable events, access to professional counseling, cognitive-behavioral therapy, and medication when necessary are factors that some physicians find beneficial in coping with emotional changes (47). Nonetheless, it is important to note that when organizations and work leaders lack the necessary skills or fail to recognize physicians’ needs, it can be difficult to implement these interventions, and they can become barriers that should be avoided (48).

It is clear that organizations and true leaders must recognize physicians as human beings who have emotions, fear, fatigue, and a need for support to carry out a profession with high physical and intellectual demands. During crises, such as infectious disease outbreaks, the constant use of personal protective equipment, frequent hand washing, daily evaluation of clinical practice guidelines, repetitive training, and long working hours per week have been associated with fatigue and neuropsychiatric symptoms in physicians (49). Therefore, it is necessary to be objective and propose programs that provide rational rest time and physical and emotional support to physicians to prevent the development of mental disorders.

Programs aimed at building resilience and helping physicians cope with problems have shown that neuropsychological feedback can also be a useful tool in supporting the social support network to organize thoughts and ideas and adapt to sudden changes during crises (50, 51). Evidence suggests that organizations should consider well-being plans for aging healthcare workers, as they are exposed to a higher number of diseases, and providing them with tools from occupational health can significantly reduce the risk of work-related illnesses (52). Undoubtedly, prioritizing the mental health of physicians and eliminating the stigma of mental health issues in these professionals, which has become a silent killer that few talks about (53, 54), is crucial. To achieve this goal, new generations of physicians must be educated, and interventions should start from medical schools, emphasizing the construction of a healthy professional lifestyle, working for the development of public policies that dignify physicians’ work and recognizing fair incentives (55). Then, suicidal ideation and suicide among physicians represent a public health issue that has received insufficient research attention. Adequate evidence needs to be generated, taking into account the specific social, occupational, cultural, economic, and health contexts in different regions of the world. Significant advances in this topic have led to the development of machine learning algorithms capable of identifying suicide-related sentiments on social media platforms (56). The utilization of this innovative tool facilitates the identification of individuals at higher risk of engaging in suicidal behavior, enabling timely intervention. Furthermore, the potential replicability of this novel approach on common or academic social media platforms within the healthcare domain can contribute to the control of suicide incidence among physicians.

In contrast to evidence published many years ago on this topic, this narrative review is the first to conduct a meta-research analysis, explicitly specifying the fulfillment or non-fulfillment of items about methodological quality. This crucial step in designing future studies aims to prevent the errors of previous studies and enhance the quality of evidence. It also provides a graphical overview of the global epidemiological distribution of mental disorders, suicidal ideation and suicide in physicians.

## 6. Conclusion

Although the available evidence is limited, it suggests that the social support network of physicians is a critical factor in determining their risk or protection from suicidal ideation and suicide. Specifically, factors such as the presence or history of neuropsychiatric symptoms, personal or family history of psychiatric disorders, suicidal ideation or suicide, high workload, experiences of discrimination and microaggressions, medical specialty (especially surgery), age, and gender (being female) may all be associated with an increased risk of suicidal ideation and suicide in physicians.

## Author contributions

NR: conceptualization, methodology, formal analysis, investigation, writing—original draft preparation, and writing review and editing. TC-B and AA: conceptualization, formal analysis, investigation, writing—original draft preparation, and writing review and editing. JR-S: conceptualization, investigation, writing—original draft preparation, and writing review and editing. IL-M: conceptualization, methodology, investigation, writing—original draft preparation, and writing review and editing. EV-J, DO, OF, and EN: investigation, writing—original draft preparation, and writing review and editing. All authors contributed to the article and approved the submitted version.

## Funding

The publication fee was financed by the Universidad Simón Bolivar, in Barranquilla - Colombia.

## Conflict of interest

The authors declare that the research was conducted in the absence of any commercial or financial relationships that could be construed as a potential conflict of interest.

## Publisher’s note

All claims expressed in this article are solely those of the authors and do not necessarily represent those of their affiliated organizations, or those of the publisher, the editors and the reviewers. Any product that may be evaluated in this article, or claim that may be made by its manufacturer, is not guaranteed or endorsed by the publisher.
